# 3D culture of neural progenitor cells in gelatin norbornene (GelNB) hydrogels: mechanical tuning and hypoxia characterization

**DOI:** 10.3389/fbioe.2025.1579580

**Published:** 2025-05-30

**Authors:** Sandra Dienemann, Ole Jacob Wohlenberg, Jan Georg Gerstenberger, Antonina Lavrentieva, Iliyana Pepelanova

**Affiliations:** Institute of Technical Chemistry, Leibniz University Hannover, Hannover, Germany

**Keywords:** gelatin-norbornene, hydrogels, IKVAV, neural stem cells, hypoxia, design of experiments, response surface methodology, 3D cell culture

## Abstract

The development of physiologically relevant three-dimensional (3D) culture platforms for neural stem cell (NSC) cultivation is essential for advancing neuroscience research, disease modelling, and regenerative medicine. In this study, we introduce norbornene-functionalized gelatin (GelNB) hydrogels crosslinked with a laminin-based peptide as a bioactive scaffold for NSC culture. A central composite design of experiments (DoE) approach was employed to systematically map hydrogel mechanical properties across varying macromer (4%–7%) and crosslinker (3–9 mM) concentrations via a response surface. This enabled precise tuning of hydrogel stiffness between 0.5 and 3.5 kPa, closely mimicking the mechanical properties of brain tissue. The optimized GelNB hydrogel formulation (5% GelNB, 8 mM crosslinker) supported NSC viability and enhanced NSC cluster formation demonstrating its suitability for 3D neural cell culture. Furthermore, we characterized the onset of hypoxia in 3D constructs using genetically encoded fluorescent hypoxia biosensors, revealing a cell density-dependent hypoxic response. At 3 × 10^6^ cells/mL, hypoxic response was detected only after 7 days of cultivation, whereas at 8 × 10^6^ cells/mL, hypoxic response was already observed within 24 h, illustrating the importance for using adequate cell numbers to avoid or achieve *in situ* physiological hypoxia. These findings highlight the importance of controlled ECM properties and oxygen microenvironments in NSC cultivation and provide valuable insights for the development of advanced biomimetic neural tissue models.

## 1 Introduction

Neural stem cells (NSCs) are self-renewing, multipotent cells that give rise to the main cell types of the nervous system, including neurons, astrocytes, and oligodendrocytes. NSCs play a vital role in nervous system development and maintenance, and their *in vitro* culture remains challenging. At the same time, developing reliable *in vitro* NSC models holds immense promise. The ability to manipulate and study NSCs in a controlled environment opens new routes for understanding neural development, unravelling disease mechanisms, and testing potential therapeutics (Wang, 2018).

Currently either animal experiments or traditional two-dimensional (2D) cell culture systems are most commonly used to study brain development and pathology (Dawson et al., 2018; Wang, 2018). While 2D cell models represent a simple, reproducible and standardizable platform in various research areas, cell adhesion on stiff glass or polystyrene surfaces forces the cells into an unnatural apical-basal polarity, restraining spreading to the x- and y-direction (Kasper et al., 2016; Kasper et al., 2021). Therefore, it becomes clear that cell-cell, as well as cell-extracellular matrix (ECM) interactions do not represent a physiological cell environment (Pampaloni et al., 2007; Baker and Chen, 2012; Kapałczyńska et al., 2016). Animal models, on the other hand, have significantly contributed to a better understanding of the pathophysiological processes of neurogenerative diseases. However, they cannot fully replicate the neuronal phenotype of human diseases (Benam et al., 2015; Dawson et al., 2018). Moreover, the implementation of the 3R principle (Replacement, Reduction and Refinement) promotes the minimization of animal testing and the development of reliable models as physiologically relevant alternatives for *in vitro* research and drug testing. Therefore, the transition towards three-dimensional (3D) *in vitro* cell culture models will enable the creation of a controllable, *in vivo* like, and physiological relevant microenvironment to bridge the gap towards established animal models while simultaneously reducing their numbers (Pampaloni et al., 2012; Dawson et al., 2018; Jensen and Teng, 2020). The mechanical properties of the cellular environment exert a significant influence on cellular processes (Ruedinger et al., 2015; Kapałczyńska et al., 2016; Lavrentieva, 2021; Lavrentieva et al., 2021; Pepelanova and Lavrentieva Antonina, 2021). Consequently, adapting the matrix composition to match the natural mechanical properties of the original tissue of the cells used is an important basis for successful 3D cultivation. While biomaterials like hydrogels allow fine-tuning of the matrix stiffness, this control is not possible in the commonly used neurospheres. The brain itself is a relatively soft tissue, with a storage modulus varying between 1 kPa and 4 kPa, depending on the brain region (Handorf et al., 2015; Kim and Choi, 2019). The cortex was found to possess a storage modulus of 1.389 ± 0.289 kPa as described by Budday et al. (Budday et al., 2015). Indeed, the exact replication of the microenvironment is essential for neural 3D cell culture, as neural cells are particularly sensitive to mechanical, structural, or topographical deviations (Bellamkonda et al., 2013). To design a multifunctional neural 3D scaffold, not only the mechanical properties, but also the incorporation of adhesion motifs, guidance structures for oriented growth and controlled release of bioactive factors have to be considered as important factors (Mahumane et al., 2018).

The chemical functionalization of gelatin through methacrylation (GelMA) is already widely used in tissue engineering and is characterized by its good biocompatibility, presence of RGD motifs and its tunability in mechanical properties (Yue et al., 2015; Pepelanova et al., 2018; Lavrentieva et al., 2019). However, a significant disadvantage of GelMA is that the photo-polymerization follows the principle of chain polymerization, where individual monomers continuously polymerize randomly. This results in a heterogeneous network within the hydrogel, alongside long polymerization times at high radical concentrations. To make photo-crosslinking faster and more cell-friendly, new functionalizations have been developed in recent years. By functionalizing gelatin with the bicyclic alkene norbornene to obtain gelatin-norbornene (GelNB), polymerization can be achieved using a thiol crosslinker. This thiol-ene reaction occurs within a few seconds between the alkene and the thiol group, following the principle of step-growth polymerization. This prevents polymerizations within the gelatin chain and results in the formation of a homogeneous network (Tibbitt et al., 2013; Mũnoz et al., 2014; Göckler et al., 2021). The choice of the crosslinker molecule, as well as its ratio to the existing norbornene groups of the GelNB, allows for a very fine-tuned adjustment of the material properties. While dithiothreitol (DTT) is most commonly used as a crosslinker, basically any molecule that contains at least two thiol groups is suitable for crosslinking. This enables the introduction of additional adhesion motifs into the hydrogel using peptide crosslinkers with terminal cysteine residues (Lin et al., 2015; van Hoorick et al., 2020). Particularly for NSCs, the presence of proteins from their natural ECM is crucial for sufficient adhesion and proliferation. For this reason, laminin, a non-collagenous, heterotrimeric glycoprotein in the basal membrane is extensively used for coating 2D culture dishes for neural cell culture. The incorporation of laminin-derived peptides into 3D hydrogel constructs is also expected to enhance the growth, differentiation and network formation of neural cells. Commonly used laminin-based motifs include the IKVAV pentapeptide situated at the C-terminus of the α1-chain, and the YIGSR sequence in the β1-chain (Iwamoto et al., 1987; Tashiro et al., 1989; Schense et al., 2000; Dalton and Mey, 2009; Li et al., 2014).

Another major limiting factor of 3D cell culture models, is the diffusion limit of oxygen, nutrients, and signal molecules towards the core of the constructs, as well as the removal of cell waste, which causes the build-up of potentially limiting gradients inside the construct (Kasper et al., 2016). On one hand, oxygen limitations can be undesirable in 3D constructs; on the other hand, the brain naturally functions at significantly lower *in situ* oxygen concentrations compared to the ambient 21% oxygen used in standard cell cultures (Zhang et al., 2011). NSC differentiation has been shown to be regulated by local oxygen levels, highlighting the necessity of physiological oxygen concentrations for reliable experimental outcomes in addition to a 3D microenvironment (Xie and Lowry, 2018). Moreover, in pathological conditions such as ischemia, tumor growth, or infection, even lower oxygen levels must be mimicked in 3D constructs. Therefore, it is essential not only to develop biocompatible, cell-supportive biomaterials for such 3D systems, but also to understand and precisely control physiological and pathological hypoxia within these constructs.

Besides direct measurements of *in situ* oxygen concentrations in the constructs, the onset of hypoxia can be visualized through the use of biosensors. In order to monitor the physiological reactions of cells to pathological or natural oxygen gradients throughout the constructs, genetically encoded fluorescence biosensors can be deployed. In this study, NSCs were transduced with the hypoxia sensor developed by Erapaneedi et al. (2016). The sensor utilizes the fluorescence protein UnaG that matures oxygen-independently and is under the same transcriptional control as the hypoxia response elements (HREs) in the genome. The production of the UnaG protein is activated following the stabilization of Hypoxia-inducible factor 1α (HIF) by hypoxia (Kumagai et al., 2013; Erapaneedi et al., 2016). In previous studies in mesenchymal stem cells, we demonstrated that hydrogel properties influence hypoxia onset and local oxygen concentrations throughout 3D constructs (Schmitz et al., 2022). We have also confirmed a quantitative correlation between the UnaG signal intensity and the prevailing oxygen concentrations (Schmitz et al., 2020; 2021; Dienemann et al., 2023).

In this study, we explored a C-IKVAV-C-peptide (IKVAV) as a crosslinker for GelNB hydrogels and assessed its impact on neural stem cell growth. A design of experiments (DoE) approach was employed to map the mechanical properties across relevant hydrogel macromer and crosslinker concentrations, enabling precise control over hydrogel stiffness. This analysis guided the selection of an optimal composition that most effectively supported NSC growth. Additionally, for the first time we characterized hypoxia onset within 3D constructs at varying NSC seeding densities, using genetically encoded hypoxia biosensors integrated into the NSCs.

## 2 Materials and methods

### 2.1 GelNB hydrogel synthesis

The GelNB hydrogel was synthesized using a modified protocol for GelNB synthesis described by Göckler et al. (2021). For the synthesis first 5-norbornene-carboxylic acid (2.680 g, 10 equiv.; Sigma Aldrich Chemie GmbH) were emulsified in 135 mL 2-(N-morpholino)ethanesulfonic acid buffer (MES-buffer; 0.5 M, pH 6; Sigma Aldrich Chemie GmbH) and activated through addition of 3-(3-dimethylaminopropyl)carbodiimide-hydrochloride (EDC-HCl; Carbolution Chemicals GmbH) (6.987 g, 5.44 mmol, 20 equiv., 22.5 mL MES) and N-Hydroxysulfosuccinimide (Sulfo-NHS; Carbolution Chemicals GmbH) (3.957 g, 2.72 mmol, 10 equiv., 11.2 mL MES). The mixture was heated to 50°C for 15 min and 6.7 g MedellaPro^®^ gelatin (Type A; Gelita), dissolved in 33.5 mL carbonate-bicarbonate-buffer, was slowly added to the mixture. The pH was set to 7.8 using 2 M NaOH and stirred overnight at 50°C. Afterwards the functionalized gelatin was diluted to 900 mL with ddH_2_O and purified by crossflow filtration (30 kDa MWCO; Sartoflow^®^, Sartorius). The purified product was frozen at −80°C and lyophilized (Alpha 2-4 LSCplus, Martin Christ).

### 2.2 Determination of degree of functionalization

To determine the DoF of the hydrogel, the amount of unreacted primary amino groups in the functionalized GelNB hydrogel was quantified via the 2,4,6-trinitrobenzenesulfonic acid (TNBS) assay and compared to unfunctionalized gelatin. For the assay, the GelNB was dissolved in carbonate-bicarbonate buffer (CB-buffer, 0.1 M) at a concentration of 1.6 mg/mL, and the gelatin was dissolved at 0.4 mg/mL (2 h, 37°C). Afterwards, each sample was mixed with 0.5 mL of 0.01% TNBS (Sigma Aldrich Chemie GmbH) and incubated at 37°C for 2 h. To stop the reaction, 250 µL of 1 M hydrochloric acid and 500 µL of 10% sodium-dodecyl sulfate were added to each sample. The absorbance was measured at 335 nm using a photometer (Eppendorf GmbH). To determine the primary amine concentrations, a glycine standard curve 0–20 μg/mL was prepared analogously to the hydrogel and the gelatin. The content of primary amines of the gelatin and GelNB were determined by comparison with the linear fit through the glycine standard curve. The DoF was then calculated using Equation 1.

$$DoF=\frac{{n\left({NH}_{2}\right)}_{gelatine\;}-{\;n\left({NH}_{2}\right)}_{GelNB}}{{n\left({NH}_{2}\right)}_{gelatine}}*100$$ (1)

Furthermore, DoF was determined also by NMR spectroscopy. The final product as well as reference MedellaPro^®^ gelatin were measured via ^1^H-NMR (600 MHz, D_2_O, Ascend 600-spectrometer, Bruker) and DoF was calculated as determined by the decrease of the lysin signal between modified and unmodified material of the same gelatin batch and normalized to the phenylalanine integral (Supplementary Figures S1, S2).

### 2.3 GelNB hydrogel preparation

The lyophilized hydrogel was dissolved in phosphate-buffered saline (PBS) in a 37°C water bath. The dissolved GelNB was supplemented with either dithiothreitol (DTT; VWR Chemicals BDH^®^), or IKVAV-peptide and DTT 2:1 (GeneCust) in PBS, and 0.1% (v/v) Lithium phenyl(2,4,6-trimethylbenzoyl)phosphinate (LAP; Tokyo Chemical Industry Co.) as photo initiator. The final hydrogel was sterilized using a pre-warmed 0.45 µm PES syringe filter. Photo-polymerization of the hydrogel was either performed in a curing box using LEDs at 405 nm or the UV-lamp of the rheometer.

### 2.4 Rheological characterization and design of experiment

For further characterization of the GelNB time-sweep tests were performed using a MCR 302 modular rheometer (Anton Paar GmbH). The storage and loss modulus of the material were measured during polymerization. For that, GelNB hydrogel was prepared as described in section 2.3 and measured directly at 37°C on a glass plate without any cells. A smooth measuring plate (∅ 20 mm, 0.6 mm gap size) was used for a sample volume of 200 µL GelNB. The photoreaction was initiated by UV lamp (365 nm, 10 mW/cm^2^) and the viscoelastic properties were monitored for a total of 240 s.

To gain a better knowledge of hydrogel composition and its influence on the hydrogel stiffness a DoE was performed. All experiments were performed with a filtered 8.02% (w/v) GelNB stock solution, 0.1 M DTT stock solution, and a 0.05 M IKVAV stock solution. A central composite design with a triplicate center point measurement and eight design points was chosen (Supplementary Table S1) for the two input factors GelNB concentration (3.98%–7.38% (w/v)) and crosslinker concentration (3.33–8.85 mM). The storage modulus *G′* of the resulting hydrogel was chosen as a single response. The design and multivariate data analysis using a multiple linear regression of the DoE was performed in MODDE 13 (Umesoft, Sartorius).

### 2.5 Cultivation of human neural progenitor cells

The immortalized human neural progenitor cell line ReNcell^®^CX were purchased from Merck. The cells originate from the cortical region of a human fetal brain, and were retrovirally immortalized through the c-myc oncogene. The cells were cultivated with serum free ReNcell^®^ NSC Maintenance Medium (Merck KGaA) supplemented with 20 ng/mL epidermal growth factor (EGF; Thermo Fisher Scientific Inc.), 20 ng/mL basic fibroblast growth factor (bFGF; Bio-Techne) and 50 μg/mL gentamicin (Merck KGaA) in laminin-coated (20 μg/mL (Bio-Techne) in DMEM/F12) cell culture T-flasks. Medium was exchanged every second day and sub-cultivation was performed when reaching 80%–90% confluency. Directly prior passaging the laminin coating has to be applied to the cell-culture plasticware by incubating the flasks for 4 h at 100 rpm on an orbital shaker in an incubator at 37°C & 5% CO_2_. For cell passaging, the medium was removed, the cells were washed with PBS and detached using accutase (Merck KGaA). Right before reseeding at 1 × 10^4^ cells/cm^2^ the laminin was removed from the fresh cell culture dish to prevent drying of the coating. Passages 5–13 were used for all experiments. To distinguish between live and dead cells Calcein-AM (Merck KGaA) and propidium iodide (Sigma Aldrich) staining was performed. For that the cells inside the hydrogel disc were incubated for 30 min.

To generate fluorescent hypoxia reporter cells, lentiviral transduction was used to introduce hypoxia biosensor constructs into the human neural progenitor cells, following the procedure outlined by Schmitz et al. (Schmitz et al., 2020). Lentiviral particles were produced using HEK-293T cells, which were seeded into TC-Scale 35 mm Petri dishes (Sarstedt AG & Co. KG) at a density of 7 × 10^5^ cells per dish in 3 mL Dulbecco’s Modified Eagle Medium (DMEM; Merck KGaA), supplemented with 3% fetal calf serum (FCS; Merck KGaA) and without antibiotics. The cells were incubated for 24 h at 37°C in a humidified atmosphere with 5% CO_2_ to reach optimal confluence for transfection. For lentiviral packaging, a mixture containing 0.83 μg pMD.G (envelope plasmid), 3.36 μg pL-HRE-dUnaG (transfer plasmid), 2.8 μg R8.91 (packaging plasmid), and 20 μg polyethyleneimine (PEI 25K; Polysciences Europe GmbH) was prepared in 500 μL OptiMEM (Thermo Fisher Scientific, Germany), incubated for 5 min at room temperature, and then added dropwise to the HEK-293T cells. After 3 h, the medium was replaced with DMEM containing 10% FCS to support viral production.

Supernatants containing lentiviral particles were collected at 24 and 48 h post-transfection and filtered through 0.45 μm syringe filters (Sartorius Stedim, Germany) to remove cell debris. For transduction, NSCs were seeded into 25 cm^2^ flasks (Sarstedt AG & Co. KG) at a density of 2 × 10^5^ cells per flask. One millilitre of filtered viral supernatant was added directly to the NSCs in culture medium, and to enhance transduction efficiency, 8 μL of 8 μg/mL polybrene solution (Sequa-brene; Merck KGaA) was included. The cells were incubated for 72 h, after which the medium was replaced, and the cells were subcultured and expanded for 2 weeks before cryopreservation at passage 10. The resulting hypoxia-reporter cells, referred to as ReNcell^®^CX-HRE-dUnaG, require the addition of 10% FCS (Merck KGaA) to the culture medium prior to microscopy and flow cytometry to ensure bilirubin-dependent maturation of the UnaG fluorescent protein.

### 2.6 Cultivation under reduced oxygen conditions in 2D

For the cultivation of the ReNcell^®^CX-HRE-dUnaG reporter cells under reduced oxygen concentrations, cells were harvested by accutase treatment, and 4 × 10^4^ cells were seeded per laminin coated well in a 24-well plate (Sarstedt AG & Co. KG). For cultivation, 1 mL of ReNcell^®^ medium supplemented with 10% FCS was added. For adherence, the cells were cultivated for 24 h at normoxic conditions (37°C, 5% CO_2_, 21% O_2_) and then transferred into a hypoxia incubator (C16, Labotect Labor Technik-Göttingen GmbH) that displaces O_2_ by N_2_ while maintaining 5% CO_2_. After either 24 h or 48 h at reduced oxygen concentrations the reporter signal was imaged via fluorescence microscopy, and subsequently, the cells were detached for flow cytometric fluorescence quantification.

### 2.7 Cell encapsulation in GelNB constructs

To encapsulate ReNcell^®^CX cells in the hydrogels, the cells were harvested using accutase treatment. Cell counting was performed using a Neubauer hemocytometer, and cells were pellets carefully resuspended in the sterile hydrogel at the concentration of 1 × 10^6^ cells/mL in 50 µL hydrogel discs and polymerized in silicone rings. Sterile silicon rings with an inner diameter of 0.6 mm were placed in the wells of a 24-well plate that serve as a mould for the hydrogel discs. A volume of 50 µL per hydrogel-disc was pipetted in the silicon mould and the hydrogel photo-polymerized immediately at 405 nm for 180 s. After curing, the silicon moulds remained in the well and 1 mL supplemented ReNcell^®^ media was added. The hydrogels were incubated at 37°C, 5% CO_2_ and 21% O_2_.

For hypoxic response experiments, the ReNcell^®^CX-HRE-dUnaG reporter cells were encapsulated in GelNB hydrogel (4.53% (w/v) GelNB, 2.59 mM DTT, 5.19 mM IKVAV; 800 Pa) at varying concentrations ranging from 1 × 10^6^ to 15 × 10^6^ cells/mL. The specified cell numbers were centrifuged (5 min, 300 xg) and the resulting pellets carefully resuspended in the sterile hydrogel prepared as described above. Sterile silicon rings with an inner diameter of 0.6 mm were placed in the wells of a 24-well plate that serve as a mold for the hydrogel discs. Per hydrogel-disc 50 μL cell-hydrogel suspension was pipetted in the silicon mold and the hydrogel cured immediately at 405 nm for 180 s. For the entire cultivation the silicon molds remained in the well and 1 mL supplemented ReNcell^®^ media with 10% FCS was added. The hydrogels were incubated at 37°C, 5% CO_2_ and 21% O_2_ for up to 7 days.

### 2.8 Live-dead staining with Calcein-AM and propidium iodide

For the microscopic assessment of cell viability, cells were stained with 4 µM Calcein-AM (Merck KGaA) and 2.5 µM propidium iodide (Merck KGaA) in ReNcell^®^ medium. The medium was removed from the hydrogel constructs, and the staining solution was added. A total of 500 µL of staining solution was used per hydrogel construct. The hydrogels were incubated with the staining solution for 30 min in the incubator before the solution was removed for fluorescence microscopy imaging.

### 2.9 Fluorescence microscopy and flow cytometry

For fluorescence microscopy, the BioTek Cytation^®^5 Cell imaging multimode reader (Agilent Technologies) was utilized. To capture the entire 3D hydrogel constructs, multiple images were taken along the x-, y-, and z-axes and assembled into one image using the montage and z-stacking functions in the Gen5 3.10 software. The TexasRed filter cube (586/647 nm) was used for propidium iodide detection, and the GFP filter cube (469/525 nm) for Calcein and UnaG.

For the fluorescence quantification of the hypoxia reporter cells, flow cytometric analysis via the BD Accuri™ C6 was performed. To separate the cells from the GelNB hydrogels, the hydrogels were digested using collagenase I (40 U/mL in Hanks Balanced Salt Solution with 3 mM CaCl_2_). Each disc was incubated for 2 h at 37°C and 450 rpm in a Thermomixer (Eppendorf SE) until a complete gelatin digestion. The remaining cells were pelleted, resuspended in 300 µL PBS and strained through a 70 μm cell strainer (Corning Inc.) for flow cytometric analysis. The green UnaG fluorescence in the gated population was excited by a Solid State Blue Laser at 488 nm and the emission detected with the integrated FL1 detector (533/30 nm).

### 2.10 Statistical analysis

Statistical analysis was performed using the OriginPro^®^2022 software (OriginLab, United States). Shapiro-Wilk test was conducted to test for normal distribution of the data. Subsequently, a one-way analysis of variance (ANOVA) was performed. Statistically significance was considered at *p* < 0.05. The design of experiments and its multivariate data analysis with a multiple linear regression was performed in MODDE 13 (Umesoft, Sartorius AG, Germany).

## 3 Results

### 3.1 Rheological GelNB characterization via design of experiment

Following the described synthesis protocol, a batch size of 6.7 g GelNB with a degree of functionalization (DoF) of 98.8% was obtained. Material properties were evaluated through rheological time sweep experiments. The stiffness and mesh size of the hydrogel are not only influenced by the macromer concentration but also by the concentration and choice of the crosslinker molecule. In order to investigate the effect of these two components on the resulting stiffness and to target specific material properties, two identical DoEs were performed using a central composite design (Supplementary Table S1). One DoE was performed with pure DTT as a crosslinker, which is a commonly used small molecule crosslinker with no inherent bioactivity (van Hoorick et al., 2020). In order to tailor the GelNB hydrogel for NSC cell culture, the bioactive motif IKVAV-dicysteine was incorporated as a crosslinker to the matrix (Yin et al., 2021). Due to its larger size, the peptide alone will not provide sufficient network stability, and was therefore combined with DTT in a 1:2 ratio in a second DoE.

**FIGURE 1:**
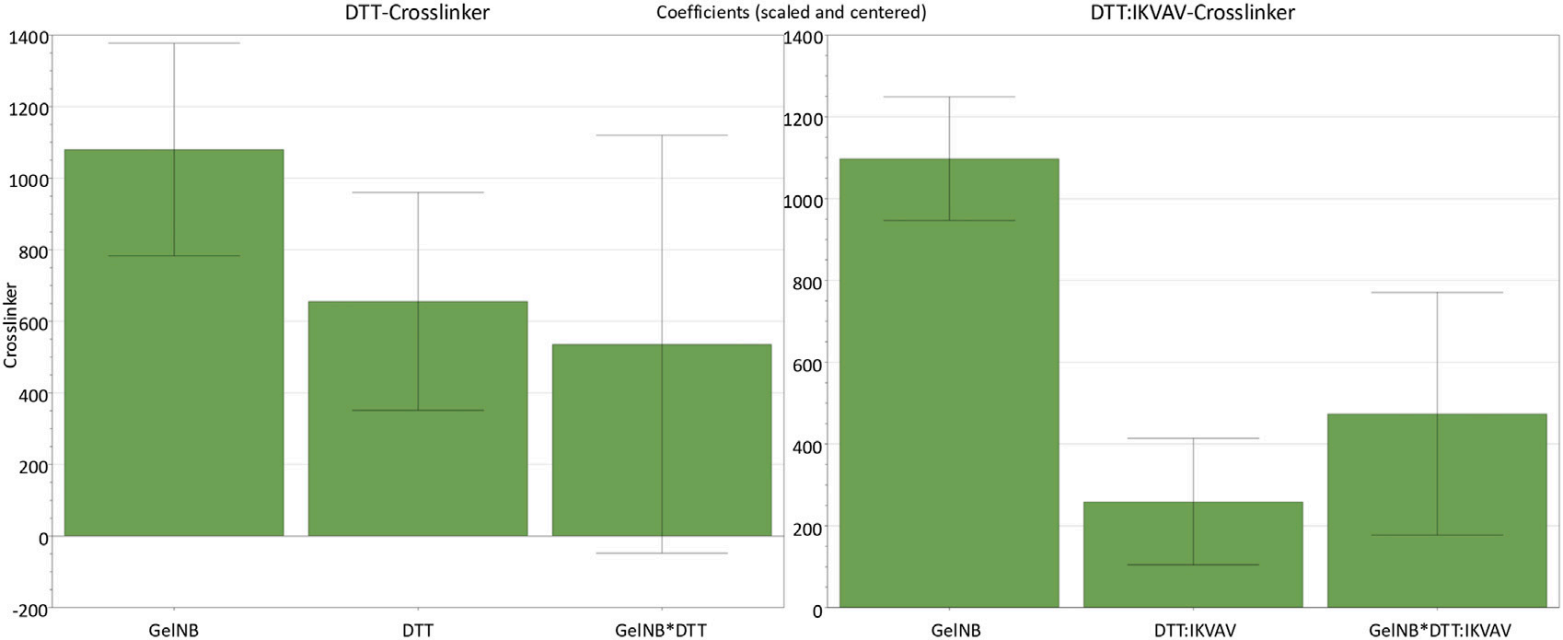
Coefficient plots for the two identical DoEs with either DTT or DTT:IKVAV as crosslinker molecule and their effects on the hydrogel stiffness response. Parameters not shown are those that included zero and therefore had no significant effect on the stiffness. GelNB [% (w/v)], DTT [mM] (n = 11, DF = 7, *R* ^2^ = 0.94), DTT:IKVAV 1:2 [mM] (n = 11, DF = 7, *R* ^2^ = 0.98). Confidence = 0.95.

In order to analyze the response as a function of the input parameters, a multiple linear regression (MLR) was performed. The effects of the input factors and their interactions were evaluated based on the regression models. Any factor that includes zero in its confidence interval was excluded due to its insignificance for the model. Resulting coefficient plots are presented in Figure 1. All the parameters for both DoEs showed positive coefficients for the prediction of the hydrogel stiffness. In both designs, the GelNB concentration (GelNB) had the strongest effect on the stiffness as a linear effect. The concentration of both crosslinkers had a weaker, but still positive influence on the hydrogel stiffness. In particular, the DTT:IKVAV mixture exerted a considerably weaker influence compared to the pure DTT. The interaction effect between gel and DTT concentration (GelNB*DTT) was not significant since it includes zero. However, it is included in the overall significant model. Notably, the GelNB*DTT:IKVAV interaction effect was significant and stronger than the linear DTT:IKVAV effect.

Furthermore, the results of the model statistics analysis are presented in the (Supplementary Table S2). The models of both DoEs were statistically significant with *p*-values below 0.001. The degree of fit of the models to the experimental data is described by *R* ^*2*^ and *R* ^*2*^_*adj*_ values between 0.911 and 0.979. The predictive power of the model is represented by *Q* ^*2*^, which also exhibits strong values of 0.787 for the DTT crosslinker and 0.938 for the DTT:IKVAV crosslinker. Therefore, both models show a high predictive power for the analysis of parameter effects as well as the reproducibility between the experiments. To visualize this, the predicted values were plotted against the experimental data (Supplementary Figures S3, S4).

**FIGURE 2:**
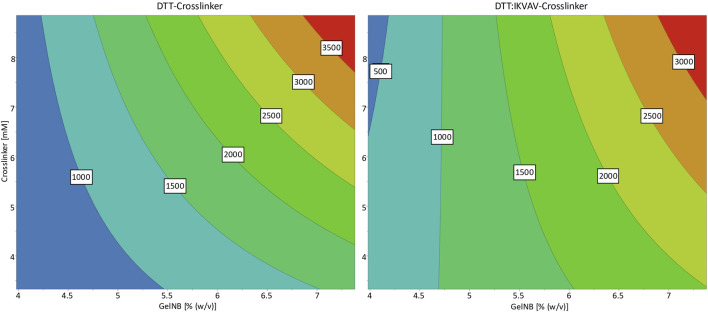
Response contour plots for the storage modulus response as a result of GelNB concentration and crosslinker concentration and choice. The contour map shows a low storage modulus in blue and a high storage modulus in red. The numbers in the contour plot represent the corresponding storage moduli (in Pa) at the individual color transitions.

Figure 2 depicts the response contour plots, which illustrate the interactions between the two input parameters and their predicted influence on the storage modulus. The contour plots for both crosslinkers exhibited a similar trend, whereby the storage modulus of the hydrogels increased with the concentration of GelNB and the crosslinkers. The pure DTT crosslinker led to overall slightly higher stiffnesses than the DTT:IKVAV crosslinker mixture. The response plots differed especially on the lower concentrations of both parameters. While the contour plot of the DTT DoE showed a clear minimum for the lowest concentrations of both GelNB and DTT, it shifted for the DTT:IKVAV DoE towards a minimal concentration of GelNB in combination with high DTT:IKVAV concentration.

While these statistical models are usually utilized for process optimizations, the results shown here served the purpose of targeting specific material stiffnesses while maintaining flexibility in hydrogel composition.

### 3.2 Identification of optimal hydrogel composition

The shown DoE results enabled the controlled variation of different hydrogel properties on the ReNcell^®^CX cells. Six different hydrogel compositions (Table 1) were tested to investigate the influence of hydrogel stiffness, crosslinker concentration and crosslinker type. The targeted storage moduli were chosen below the physiological stiffness of the grey matter of 1.389 ± 0.289 kPa (Budday et al., 2015) in the brain. The NSCs were encapsulated in the respective hydrogel at 1 × 10^6^ cells/mL GelNB and 50 µL hydrogel discs were polymerized. The discs were incubated at 37°C, 5% CO_2_ for either three or 7 days.

**TABLE 1:** Calculated GelNB hydrogel compositions based on DoE model resulting in a storage modulus of 600 Pa, 800 Pa and 1090 Pa that were tested with ReNcell^®^CX NSCs. LAP 0.1% (v/v).

Hydrogel composition	Storage modulus *G’* [Pa]	GelNB [% (w/v)]	DTT [mM]	IKVAV [mM]	Ratio [mM thiol: % GelNB]
1	800	4.539	4.214	-	1.857
2	800	4.528	2.592	5.185	3.435
3	800	4.564	2.896	5.793	3.808
4	800	4.155	1.342	2.684	1.938
5	1090	4.855	2.114	4.228	2.613
6	600	4.104	1.965	3.930	2.873

**FIGURE 3:**
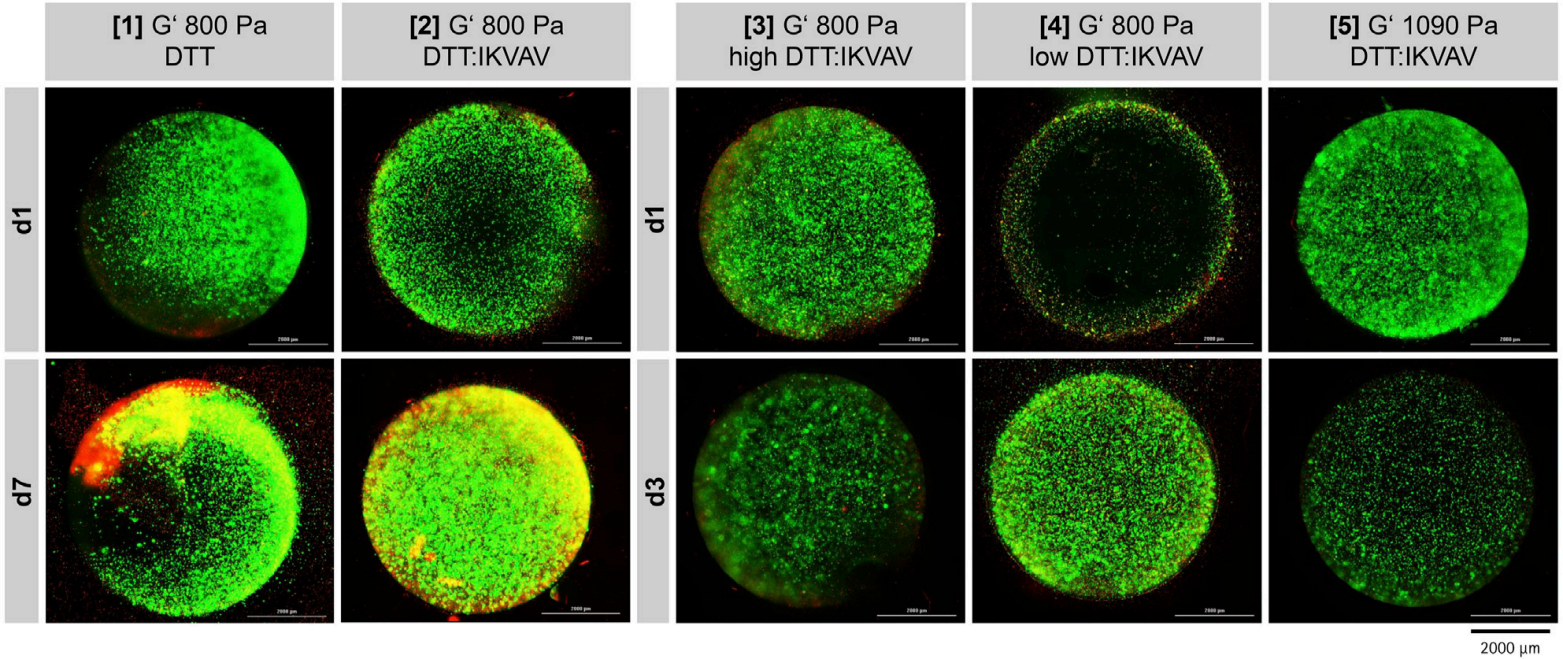
Fluorescence microscopic images ReNcell®CX cell viability after one and 7 days of cultivation (37°C, 5% CO_2_) in five different GelNB hydrogel compositions (Table 1). “high DTT:IKVAV” refers to a high crosslinker concentration, “low DTT:IKVAV” to a low crosslinker concentration. Encapsulated cell concentration 1 × 10^6^ cells/mL. Green: Calcein-AM staining (viable cells), Red: Propidium iodide staining (dead cells).

At the respective time points the cells were stained with Calcein-AM and propidium iodide to evaluate the cell viability inside the different hydrogel compositions. The fluorescence signals were evaluated via fluorescence microscopy montage in x-, y- and z-axis (Figure 3). Hydrogel composition 6 is not presented, as its structural integrity was insufficient, leading to complete dissolution within 24 h of incubation.

First, the cell viability after one and 7 days in either GelNB:DTT hydrogel (Table 1, composition 1) or GelNB:DTT:IKVAV (Table 1, composition 2) was assessed. The cells in both hydrogel compositions demonstrated a good viability during the entire cultivation time of 7 days (Figure 3, [1] and [2]). A clear proliferation of the cells and establishment of small clusters within the gel could be observed for the GelNB:DTT:IKVAV hydrogel composition 2. Based on this observation, the combination of DTT and the IKVAV-peptide crosslinker was chosen for subsequent experiments.

To evaluate the influence of GelNB-to-crosslinker ratio on cell growth and viability the hydrogel compositions 3 and 4 (Table 1) were tested over 3-day cultivation period. Both compositions predominantly showed viable cells after 3 days of cultivation, with composition 4 exhibited higher proliferation compared to composition 3.

In composition 5 a higher storage modulus of 1090 Pa compared to the previous compositions was tested. NSC cultivation in this composition led to a lower Calcein-AM signal compared to composition 3 and 4.

Overall, further modifications of the GelNB-to-crosslinker ratio did not enhance cell proliferation within the hydrogels; therefore, composition 2 was selected for subsequent experiments.

### 3.3 Evaluation of NSCs hypoxic response in 2D cell culture

**FIGURE 4:**
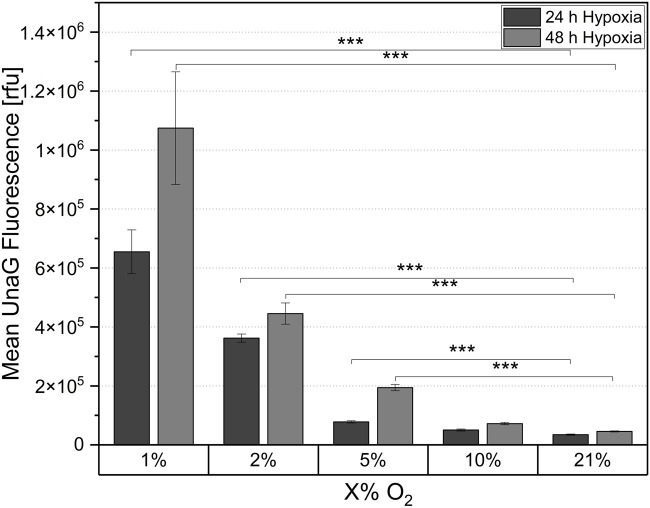
Mean UnaG fluorescence of the ReNcell®CX-HRE-dUnaG hypoxia reporter cells in 2D cell culture after 24 h and 48 h cultivation in different oxygen concentrations (1%, 2%, 5%, 10%, 21%). Mean UnaG fluorescence determined via flow cytometry. Mean n = 3 ± SD; ***p < 0.001.

The *in vivo* oxygen availability of cells is usually highly dependent on the originating tissue and is defined by a balance of oxygen supply and consumption. Therefore, it can be assumed that the onset of the hypoxic response mediated by the stabilization of HIF-1α usually correlates with the natural biological hypoxic niche (Ivanovic, 2009; Carreau et al., 2011; Bahsoun et al., 2018). To determine the oxygen concentration that triggers hypoxia onset in NSCs and quantify the fluorescence signal which corresponds to defined local oxygen levels, cells were cultivated first under reduced oxygen concentrations (1%, 2%, 5%, 10%, and 21% O_2_) in 2D for either 24 h or 48 h in laminin-coated 24-well plates. The sensor signal was visualized via fluorescence microscopy (Supplementary Figure S5) and quantified by flow cytometry (Figure 4).

After 24 h of cultivation at both 1% and 2% oxygen concentration, a significant reporter signal indicating hypoxia onset was quantified using flow cytometry (Figure 4). The signal was strongest at 1% pO_2_, and was approximately 38% lower at 2% pO_2_. After 24 h of cultivation at 5% pO_2_, only a minimal reporter signal was observed compared to the normoxic (21% pO_2_) control, with the signal approaching the control value as the oxygen concentration was increased to 10% pO_2_. Therefore, the threshold for initiating a hypoxic response via HIF-1α stabilization in the NSCs used in this study was between 2% and 5% pO_2_. In comparison to 24 h cultivation, the fluorescence intensity of the reporter cells was higher after 48 h incubation under reduced oxygen (Figure 4). Specifically, the fluorescence intensity increased 1.6-fold after 48 h of cultivation at 1% O_2_ compared to the measurements after 24 h. An increase in the reporter signal was also observed at 2% and 5% pO_2_, although the proportional increase was smaller. After 48 h of incubation at 10% pO_2_, no increase in fluorescence intensity compared to the controls was observed. This suggests that 10% ambient oxygen did not induce a cellular response in the cells. For both hypoxic cultivation times the fluorescence signal was clearly visible in the fluorescence microscopic images at 1% O_2_ (Supplementary Figure S5). For the higher tested oxygen concentrations, the signal was significantly weaker but correlated with the flow cytometric fluorescence quantification. After 24 h measured relative fluorescence of cells at 1% oxygen concentration was 6.55 × 10^5^ rfu, for 2% - 3.32 × 10^5^ rfu, for 5% - 7.80 × 10^4^ rfu.

### 3.4 Oxygen availability in 3D GelNB hydrogel constructs

**FIGURE 5:**
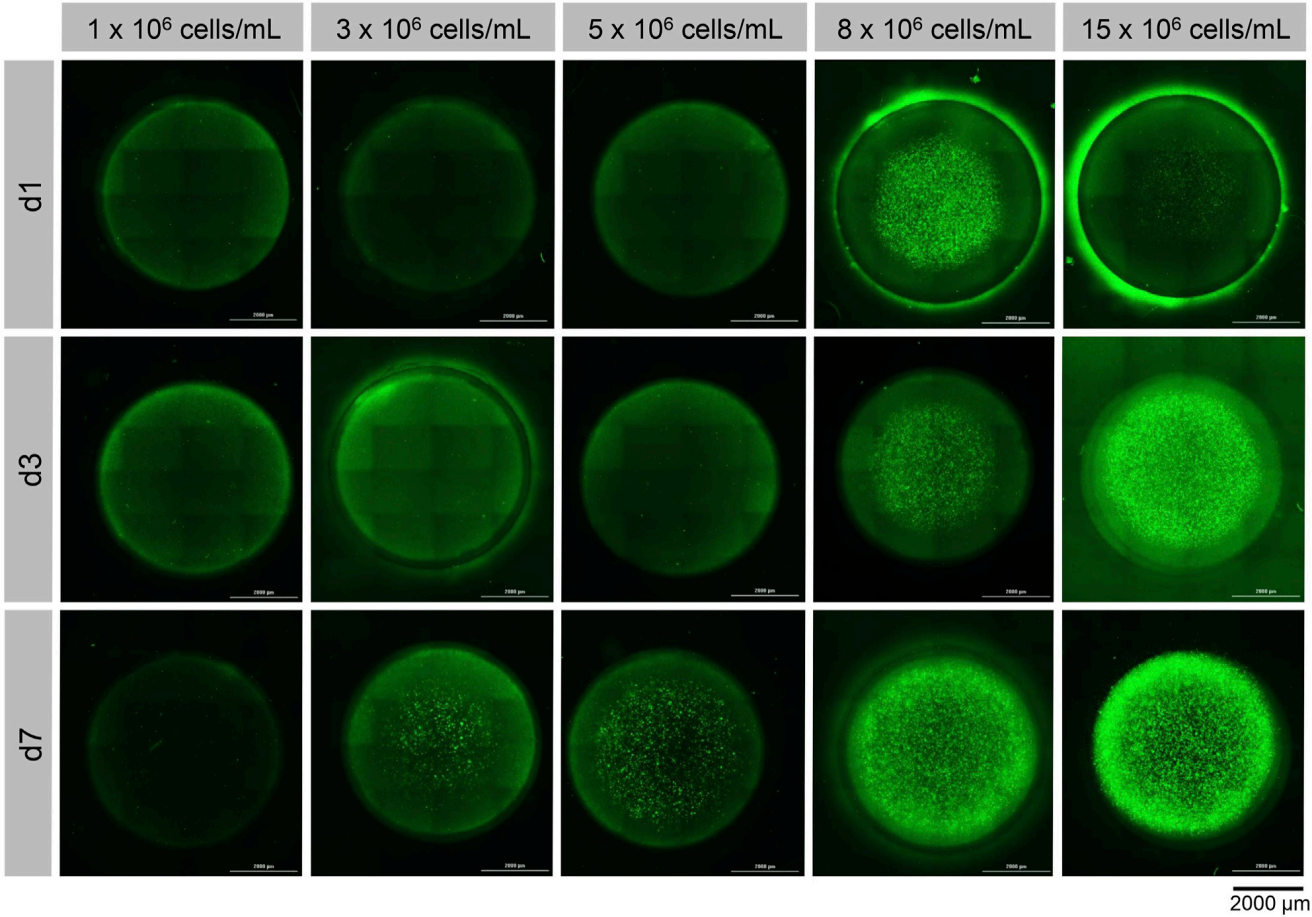
Visualization of hypoxic response of ReNcell®CX-HRE-dUnaG hypoxia reporter cells using cell concentrations ranging from 1 × 10^6^ to 15 × 10^6^ cells/mL in GelNB hydrogel composition 2 (4.53% (w/v) GelNB, 2.59 mM DTT, 5.19 mM IKVAV; 800 Pa) after 1-, 3- and 7-days incubation at 37°C and 21% O_2_.

To investigate the onset of hypoxia within the 3D constructs at high cell densities, hypoxia reporter NSCs were encapsulated at different cell concentrations (1, 3, 5, 8, 15 × 10^6^ cells/mL) in the hydrogel composition which demonstrated the highest cell viability (Table 1; composition 2). The 50 µL discs were incubated under ambient oxygen concentrations (21% O_2_) for a total of 7 days. The hypoxia reporter signal, that corresponds with the cells reaction to reduced oxygen concentrations, inside the construct was evaluated after one, three and 7 days via fluorescence microscopy and flow cytometry. The fluorescence microscopic images (Figure 5) demonstrate a clear increase in fluorescence signal in relation to the cell concentration and cultivation time.

For the lowest tested cell concentration of 1 × 10^6^ cells/mL, a reporter signal could only be detected in few cells after 7 days of cultivation. Hydrogel discs with concentrations of 3 × 10^6^ and 5 × 10^6^ cells/mL showed a clear fluorescence signal in the center of the hydrogel disc at day seven, but no fluorescence at previous time points. At higher cell concentrations of 8 × 10^6^ cells/mL and 15 × 10^6^ cells/mL a clear sensor signal was detected already after 1 day of cultivation, which increased steadily in both hypoxic core and intensity over the 7-day cultivation period.

**FIGURE 6:**
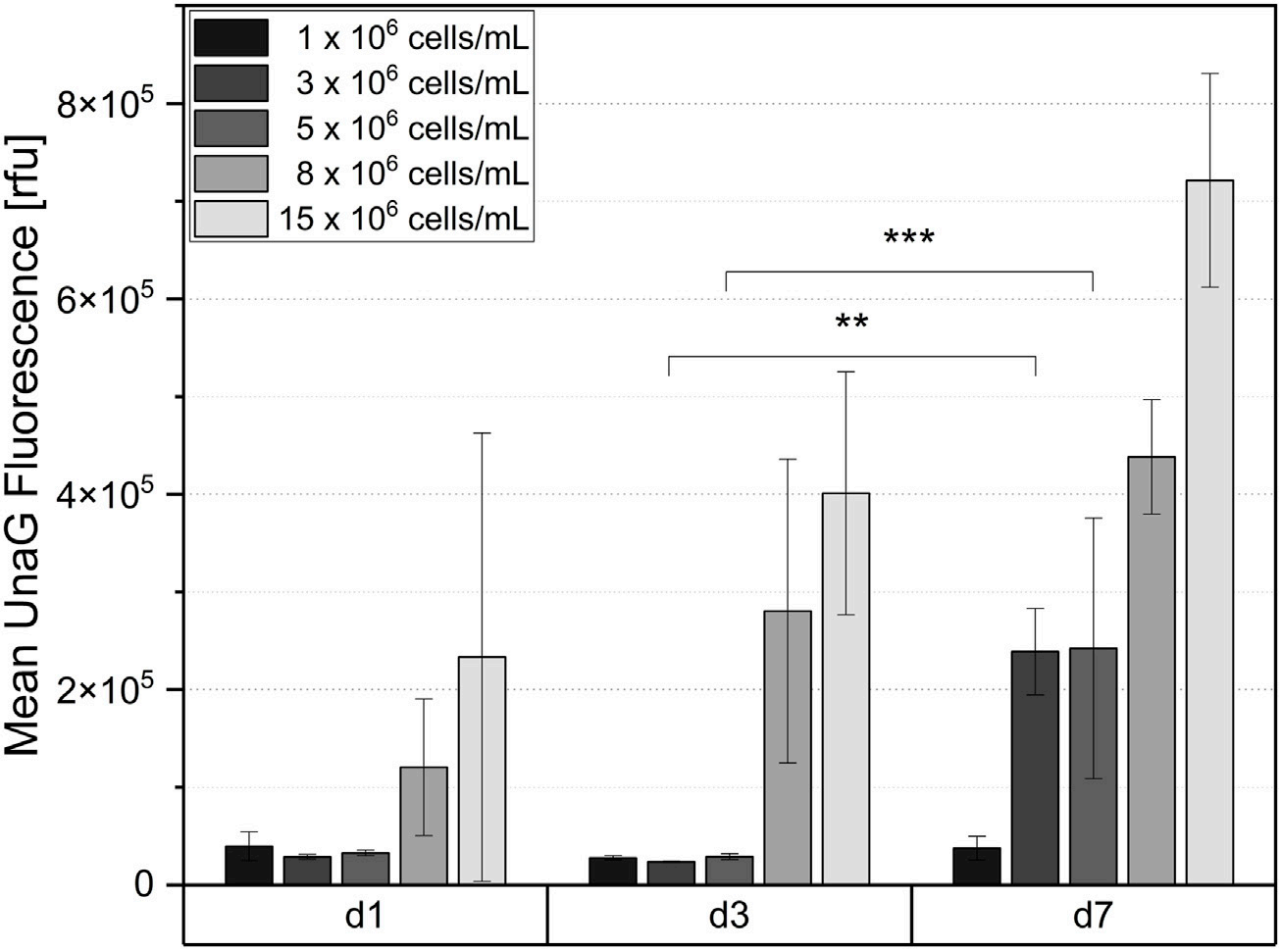
Mean UnaG fluorescence intensity of ReNcell®CX-HRE-dUnaG reporter cells after 1-, 3- and 7-days cultivation in GelNB hydrogel (4.53% (w/v) GelNB, 2.59 mM DTT, 5.19 mM IKVAV) using cell concentrations ranging from 1 x 10^6^ to 15 x 10^6^ cells/mL. Flow cytometric measurement after collagenase digestion of hydrogel. Mean n = 3 ± SD; **p < 0.01, ***p < 0.001.

Subsequently to the microscopic imaging, the hydrogel discs were dissociated by collagenase treatment and the fluorescence intensity of the UnaG was quantified by flow cytometric analysis. The quantification of the fluorescent signal confirmed that there was no significant difference in fluorescence intensity at the lowest level of cell concentration (1 × 10^6^ cells/mL) after 7 days of cultivation, indicating that cells do not experience hypoxia. Significant signal was detected only after a week of cultivation for cell densities of 3 × 10^6^ and 5 × 10^6^ cells/mL. Thus, the quantitative results obtained by flow cytometry (Figure 6) strongly correlate with the microscopy results (Figure 5). The sustained elevation of the hypoxia signal throughout the cultivation time indirectly suggests increased oxygen consumption due to the cell proliferation, and further demonstrates that the hydrogel constructs function as a dynamic system, supporting continuous cell growth for at least 1 week.

## 4 Discussion

This study aimed to establish a biohybrid GelNB hydrogel platform for the cultivation of human neural cortical stem cells (ReNcell^®^CX). For this purpose, lysine residues of the gelatin were functionalized with norbornene and the DoF was characterized via ^1^H-NMR and TNBS as a fully functionalized material of >98%. The mechanical properties of GelNB in combination with two different crosslinker molecules (DTT, DTT:IKVAV) were characterized via rheology in the course of a two-factor DoE approach in a central composite design. While DoE is commonly used for process optimization, it has also been used to study how multi-component biomaterials can be optimized in their formulation, including how the individual components influence each other in terms of stiffness or biocompatibility. For example, a DoE approach was used to study how cryogels of collagen-chitosan-fucoidan can be best manufactured (Carvalho et al., 2022). And in an example more relevant for neural cell culture, Lam et al. (2015) used a DoE approach to optimize the formulation of a hydrogel for the cultivation of neural progenitor cells based on hyaluronic acid, RGDs and laminin-based ligands. These studies highlight the advantages of applying a DoE approach in biomaterial formulation optimization. DoE enables the simultaneous investigation of multiple variables, leading to significant savings in time and resources. Unlike traditional one-factor-at-a-time methods, it uncovers synergistic or antagonistic interactions between components that might otherwise go unnoticed. Importantly, DoE yields a predictive model that links input parameters to desired material properties, facilitating targeted formulation design. In our case we could show that GelNB macromer concentration is the dominant factor affecting hydrogel stiffness, and that the DTT:IKVAV crosslinker displays weaker contribution to stiffness compared to pure DTT, probably related to its bulkier molecular structure.

Typical thiol-based linkers used for GelNB crosslinking include DTT, multiarm PEG-thiols (Arkenberg et al., 2022) and thiolated gelatin (Tytgat et al., 2019) or hyaluronic acid (Shih et al., 2016). The integration of extracellular matrix (ECM) materials or motifs as crosslinkers can be used to engineer cell responsiveness and specific bioactivity into the GelNB hydrogels. To adapt the GelNB matrix for NSC cultivation, we used the bifunctional C-IKVAV-C peptide from laminin with the aim to improve neural cell adhesion and growth inside the hydrogel. The use of bi-thiolated bioactive peptides as a crosslinker for GelNB has not been extensively studied in the literature so far, probably related to the fact that they create very soft hydrogels, when used as the sole crosslinker. We circumvented this problem by adding small amounts of DTT, leading to a complex network architecture and effects, as shown by the significant interaction between GelNB:DTT:IKVAV of the DoE. We also performed experiments with YIGSR-dicysteine as a laminin-derived crosslinker for GelNB. Hydrogels created with this crosslinker were softer and were unstable for long-term NSC cultivation (Supplementary Table S2; Supplementary Figure S6). Possible differences in the conformation between IKVAV (Yamada et al., 2002) and YIGSR (Bella et al., 2009) might explain why they yield GelNB hydrogels of different stability. All experiments in this study were therefore solely performed with IKVAV peptide in the crosslinker.

Based on the statistical DoE calculations, the tunability of the GelNB platform could be utilized to a full extent. A desired hydrogel stiffness mirroring neural tissue was selected and various hydrogel compositions were tested for NSC 3D cultivation. We could show that hydrogels containing IKVAV as a crosslinker were better able to support NSC proliferation and cluster formation, compared to GelNB hydrogels of identical stiffness, but crosslinked with DTT only. Next, we continued to maintain the hydrogel stiffness, while introducing a variation in the thiol/GelNB ratio. A comparison was performed between cultivations of 2 mM thiol/% GelNB and 4 mM thiol/% GelNB in hydrogel discs of the same stiffness and using the same crosslinker DTT:IKVAV. Although we expected that adding more bioactive linker will result in enhanced cell proliferation, we observed that NSCs were growing better in the lower thiol/norbornene ratio. The reason for this is probably related to the fact that the higher thiol-to-norbornene ratio results in a slightly more crosslinked, denser and less cell-permissive hydrogel. Based on the microscopy evaluation, a sweet spot of 3 mM thiol/% GelNB was identified as being optimal in providing sufficient bioactive linker and also creating favorable network architecture for the NSCs at a stiffness of 800 Pa. Keeping this crosslinker ratio, but increasing or decreasing the stiffness did not improve on the cultivation. A lower stiffness of 600 Pa resulted in soft hydrogels of poor integrity upon prolonged cultivation, while stiffer hydrogels led to denser networks, which did not support cell growth as well.

The thiol-to-norbornene ratio controls the extent of the click reaction, with a stoichiometric ratio of 1:1 resulting in complete crosslinking related to a uniform, stable hydrogel network. Excess thiol leads to unreacted thiols which may alter bioactivity, especially when using highly reactive molecules like DTT, and excess norbornene yields a softer, more porous hydrogel. It is difficult to know the exact stoichiometric ratio of GelNB, as it is an undefined material of natural origin, consisting of a mixture of molecular weights. Some assumptions can be made based on average molecular weight of gelatin A and the DoF, but they remain approximations. Many groups circumvent this problem by modifying gelatin as both a norbornene and a thiol partner, resulting in easier adjustment of an ideal 1:1 stoichiometric ratio (Göckler et al., 2021). Working at mM/% GelNB ratio of three like in our case, has been speculated to result in complete crosslinking by some researchers (Mũnoz et al., 2014). The GelNB hydrogel system allows an elegant tuning of mechanical matrix properties through independent adjustment of macromer concentration and/or crosslinker ratio. Further studies are needed to explore how the individual GelNB components may influence NSC behavior. The effect of matrix stiffness on neural stem cell differentiation has been studied mostly in 2D on coatings of various hydrogel systems such as: on methacrylamide chitosan surfaces (Leipzig and Shoichet, 2009), PEG-RGD hydrogels (Stukel and Willits, 2018) or variable moduli interpenetrating polymer networks (Saha et al., 2008). These studies point out that differentiation into neurons is promoted on softer matrices (<1 kPa), while glial fate tends to occur on stiffer materials (1–10 kPa).

The degradation of the GelNB hydrogel was also not investigated directly, as this has been already extensively studied by many other research groups (Göckler et al., 2021; Van Damme et al., 2021). Findings demonstrate that enzymatic digestion occurs by bulk or surface erosion mechanism depending on the crosslinking density (Greene and Lin, 2015). The structural integrity of the developed GelNB platform was shown to be stable over 7 days of NSC cultivation, even with variable cell numbers encapsulated. This suggests that cellular remodeling is minimal at such time frames. In contrast, active enzymatic digestion with collagenase was used to release cells for analysis, with constructs being completely digested within 2 h of treatment.

Both natural and synthetic hydrogels have been used with success for 3D cell culture of neural stem and progenitor cells. Natural materials used for culturing include collagen, hyaluronic acid (Seidlits et al., 2019), Matrigel and fibrin (Layrolle et al., 2023). Matrigel is especially popular for brain organoid culture as it is rich in ECM proteins (laminin, collagen IV) and can support long-term cultures (Lancaster and Knoblich, 2014). However, it has an undefined composition and it is tumour-derived, making potential translation a somewhat tricky issue. Natural materials need to be frequently chemically modified to ensure better control over mechanical properties. In addition, batch-to-batch consistency can fluctuate due to sourcing from natural materials. For this reason, synthetic hydrogels based on self-assembling peptides (Gao et al., 2017) and elastin-like peptides (Madl et al., 2019) have also been extensively explored for 3D neural cell culture. These also have to be modified with laminin motifs, RGDs or protease recognition sites to ensure bioactivity, biodegradability and cell adhesion. Synthetic materials like peptide hydrogels possess reproducible and controllable properties but they can be costly to scale, their gelation mechanisms tend to be in the range of minutes at their fastest (Chen et al., 2014) thus hindering integration into automated platforms, and their novelty makes their regulatory and clinical translation potential uncertain. GelNB is to be classified as a semi-synthetic hydrogel (Ruedinger et al., 2015). Like shown in the current study, its advantages include biocompatibility, degradability, and the ability to finetune its mechanical properties. Bioactive motifs can be easily introduced as the thiolated crosslinker. The photopolymerization occurs in the manner of seconds and it yields transparent hydrogels. The production process is simple, cost-efficient and scalable. At the same time, it should be emphasized that the gelatine component in GelNB is derived from animal sources and is likely to show batch variabilities related to sourcing. This problem can be circumvented by the use of recombinant gelatines (Schlauch et al., 2024), which would of course make the final product more costly.

It is important to note that our assessment of cell viability and clustering within the GelNB hydrogels is based on qualitative analysis of live/dead staining. To gain a more comprehensive understanding of the interactions between NSCs and the hydrogel matrix, future studies should incorporate quantitative metabolic assays and advanced cell tracking techniques. These approaches will allow for more precise evaluation of cell behavior, viability, and dynamic responses within the 3D environment.

Suitable mechanical properties and 3D architecture alone are not enough to accurately replicate a physiological microenvironment *in vitro*. Other critical factors, such as the local oxygen concentration, must also be carefully considered to ensure a biologically relevant system. Indeed, *in vivo* oxygen tensions vary widely in different healthy tissues, and in case of infection or disease (e.g., tumor growth), *in situ* oxygen concentrations can drop to pathologically low levels (below 1% or 8 mmHg O_2_) (Krock et al., 2011; Ortega et al., 2017; Fathollahipour et al., 2019). Although the brain does not perform mechanical work, unlike skeletal muscles or the heart, it is one of the organs with the highest metabolic activity in the body (Rink and Khanna Abstract, 2011). In the brain, oxygen consumption exhibits significant regional variability and dynamic regulation, with grey matter displaying higher oxygen demand than white matter (Pantano et al., 1984). Oxygen concentration in the brain is measured to be as low as 11.4–53.2 mmHg (corresponding to 1.5%–7.0% O_2_) and even lower in the embryonic brain (0.076–7.6 mmHg or 0.01%–1.0% O_2_) (Zhang et al., 2011).

Despite having gained widespread acceptance in the scientific community, 3D culture systems, including neuronal models, lack reproducibility and often the data obtained by different research groups are contradictory. One possible reason for this variability is the significant differences in the size of the 3D *in vitro* constructs and the cell densities used in the different protocols. This results in different local oxygen levels, which in turn influence the cellular responses. While in 2D culture systems, oxygen tensions mainly depend on the oxygen concentration in the incubator and the oxygen diffusion from the air into the cell culture medium and cells, the increasing geometric complexity and higher cell densities in 3D systems lead to the formation of local hypoxic regions. Thus, new approaches to monitor and control oxygen concentrations in 3D cultures, as well as cell responses to these concentrations are urgently needed.

To further investigate the developed 3D GelNB hydrogel-based NSC cultivation systems, we incorporated genetically encoded hypoxia biosensors into the cells, allowing non-invasive monitoring of hypoxia onset within the constructs over time. In our previous studies, we have shown that these sensors are a reliable tool that allows to quantitatively and cell-specifically monitor the onset of hypoxia in different cell types (Schmitz et al., 2020; 2021; 2022; Dienemann et al., 2023; Fleischhammer et al., 2024). The genetically encoded biosensors used in this study detect the stabilization of HIF-1α, a key regulator of the cellular response to hypoxia. More than 300 target genes are regulated by this transcription factor, and the onset of hypoxia represents a complete change in cellular state and behavior. NSCs with integrated hypoxia reporter (ReNcell®CX-HRE-dUnaG) encapsulated in GelNB constructs at varying cell densities, ranging from 1 × 10^6^ cells/mL to 15 × 10^6^ cells/mL, demonstrated a cell density-dependent increase in reporter fluorescence over a cultivation period of 7 days. Both microscopic analysis and flow cytometry demonstrated that during cultivation, hypoxia increased in hydrogel constructs, indirectly indicating that cells were proliferating within the GelNB hydrogels.

To indirectly estimate the oxygen concentrations to which cells are exposed in GelNB constructs at different cell densities and time points, we cultured the same cells under controlled oxygen levels in 2D and measured the resulting population fluorescence using flow cytometry. Our analysis revealed that the general threshold for the hypoxic response of NSCs occurs at approximately 5% oxygen concentration in 2D cultures. Comparing relative measured fluorescence in 2D and 3D, we can speculate that seeding densities of 8 × 10^6^ cells/mL and 15 × 10^6^ cells/mL result in *in situ* hypoxia similar to 2%–5% oxygen tensions. Several studies have reported the effects of reduced oxygen tensions on NSCs. (Xie and Lowry, 2018). Ortega et al. (Ortega et al., 2017) demonstrated that oxygen levels at 1% or lower reduce cortical NSC proliferation rates, whereas an oxygen tension of 3% positively influences proliferation in 2D cultures. In contrast, fetal ventral mesencephalic NSCs did not exhibit changes in their proliferation rate but showed a decrease of apoptosis when cultured at 3% oxygen tension under 2D conditions over a 3-day period (Santilli et al., 2010). Moreover, if cultivated in 3D neurospheres, mesencephalic NSCs demonstrated a significant increase and forebrain cells a mild increase in proliferation under 3% oxygen (Storch et al., 2001). Regarding differentiation, mild hypoxia (2.5%–6% O_2_) is believed to enhance neurogenesis and oligodendrogenesis, whereas severe hypoxia (1%–2% O_2_) promotes astrogliogenesis (Xie and Lowry, 2018). Our study, for the first time, systematically evaluated the hypoxic response of NSCs within GelNB hydrogel constructs, providing insights into how different cell densities and local oxygen concentrations influence this response, which can lead to better control in experimental setups.

There are no uniform protocols regarding cell densities used in 3D hydrogel constructs. For example, Zhou et al. (2020) encapsulated a mixture of NSCs and bone mesenchymal stem cells with a total cell density of 10 × 10^6^ cells/mL in GelMA constructs similar in size to our study. They reported that GelMA scaffolds together with bone mesenchymal stem cells promoted neuronal differentiation while reducing astrocyte formation. In agreement with the literature and our findings, this effect could be attributed to the induction of mild hypoxia. However, many research groups employ a relatively low number of cells for encapsulation. For example, Arkenberg et al. (2022) encapsulated iPSCs in GelNB at the density of 2 × 10^6^ cells/mL and demonstrated successful differentiation into neuroectoderm. An even lower cell number (1.67 × 10^5^ cells/mL) of NSCs was used by other researchers for encapsulation in self-assembling peptide hydrogels (Cheng et al., 2013). In our study, we observed that such low cell densities fail to establish a physiological microenvironment, as even at a density of 1 million cells per mL, no increase in the hypoxia reporter signal was detected over the 7-day cultivation period. Our results also suggest that to achieve *in situ* oxygen concentrations close to or below 1%, cell densities higher than 15 million cells per mL must be used, or the oxygen concentration must be adjusted using hypoxia chambers or incubators.

In summary, our study demonstrates that incorporation of IKVAV peptide improves the viability and proliferation of NSCs in GelNB:DTT hydrogels. By applying a Design of Experiment (DoE) approach, we systematically characterized the storage moduli of different GelNB and crosslinker combinations. The optimal composition determined in this study is a GelNB to crosslinker ratio of 1:3 with a storage modulus of 800 Pa, which provides a suitable balance between mechanical support and biofunctionality for NSC culture. In addition, for the first time, we present a direct monitoring of NSCs response to hypoxia in GelNB hydrogel constructs, assessing both spatial and temporal dynamics. NSCs with integrated genetically encoded hypoxia biosensors, demonstrated an increase in fluorescence over the cultivation period at all cell densities above 1 million cells per mL. This increase indirectly indicated cell proliferation within the hydrogel constructs over the 7-day cultivation period. Furthermore, these findings suggest that once seeded with cells, 3D hydrogels function as dynamic systems with continuously changing microenvironments. To induce hypoxia in GelNB hydrogels with 800 Pa stiffness, using 5 mm hydrogel discs of 50 μL, cell densities higher than 5 × 10^6^ cells/mL are required. A density of 1 × 10^6^ cells/mL for NSCs does not generate a hypoxic environment within these 3D constructs. These findings contribute to a better understanding of NSC behavior in GelNB-based 3D models, highlighting how critical experimental setups, particularly cell densities, are influencing cellular responses and onset of *in situ* hypoxia.

## Data Availability

The raw data supporting the conclusions of this article will be made available by the authors, without undue reservation.
