# Supplementary material for: 3D culture of neural progenitor cells in gelatin norbornene (GelNB) hydrogels: mechanical tuning and hypoxia characterization
Source: Front Bioeng Biotechnol. 2025 May 30;13:1579580. doi: 10.3389/fbioe.2025.1579580 (PMC12163324; doi:10.3389/fbioe.2025.1579580)
Supplement: Supplementary file 1 [file DataSheet1.pdf]

Supplementary Material

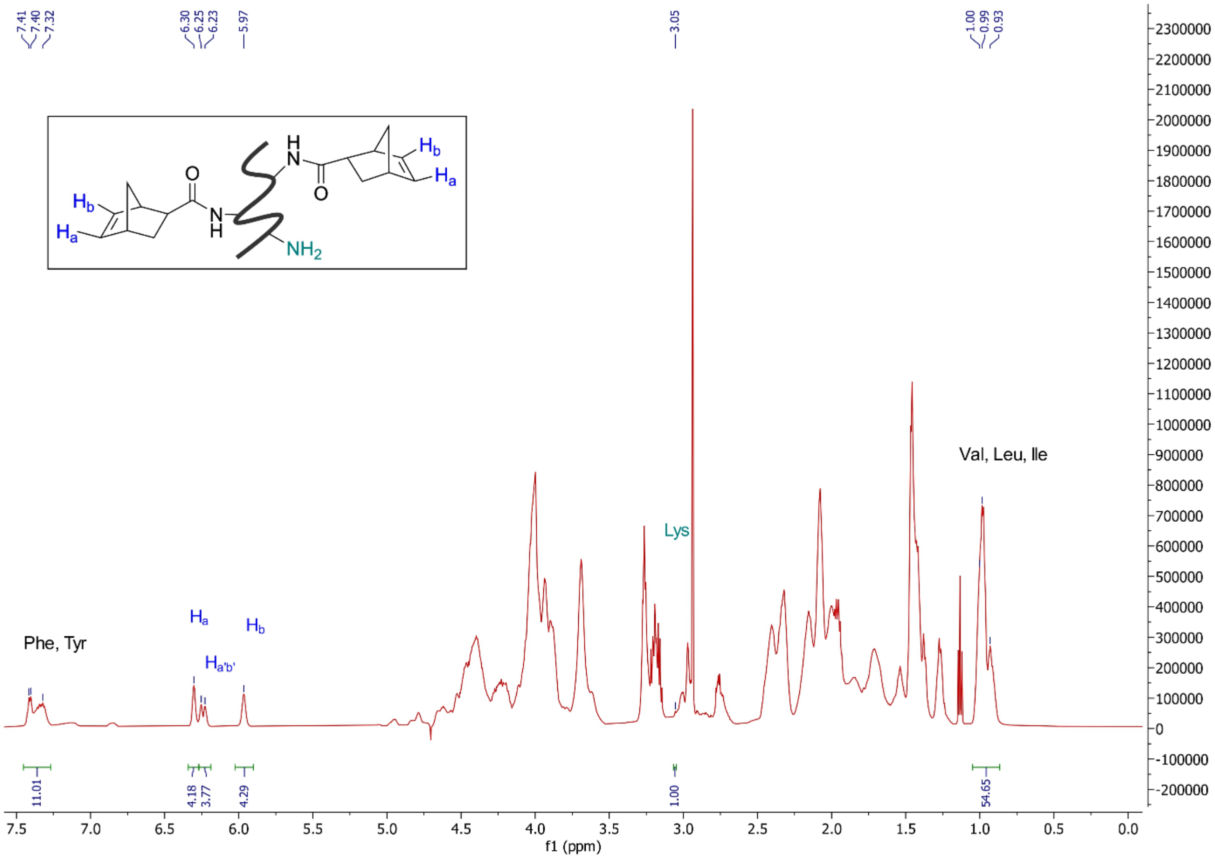

Figure S1: ^{1}H-NMR spectrum of the functionalized GelNB hydrogel. (600 MHz, D2O) δ:: 7.41 – 7.32 (m, Phe, Thr), 6.30 (m, -CHa=CHb-), 6.24 (m, -CHa'=CHb'-), 5.97 (m, -CHa=CHb-), 3.07 – 3.04 (m, Lys), 1.00 – 0.93 (m, Val, Leu, Ile).

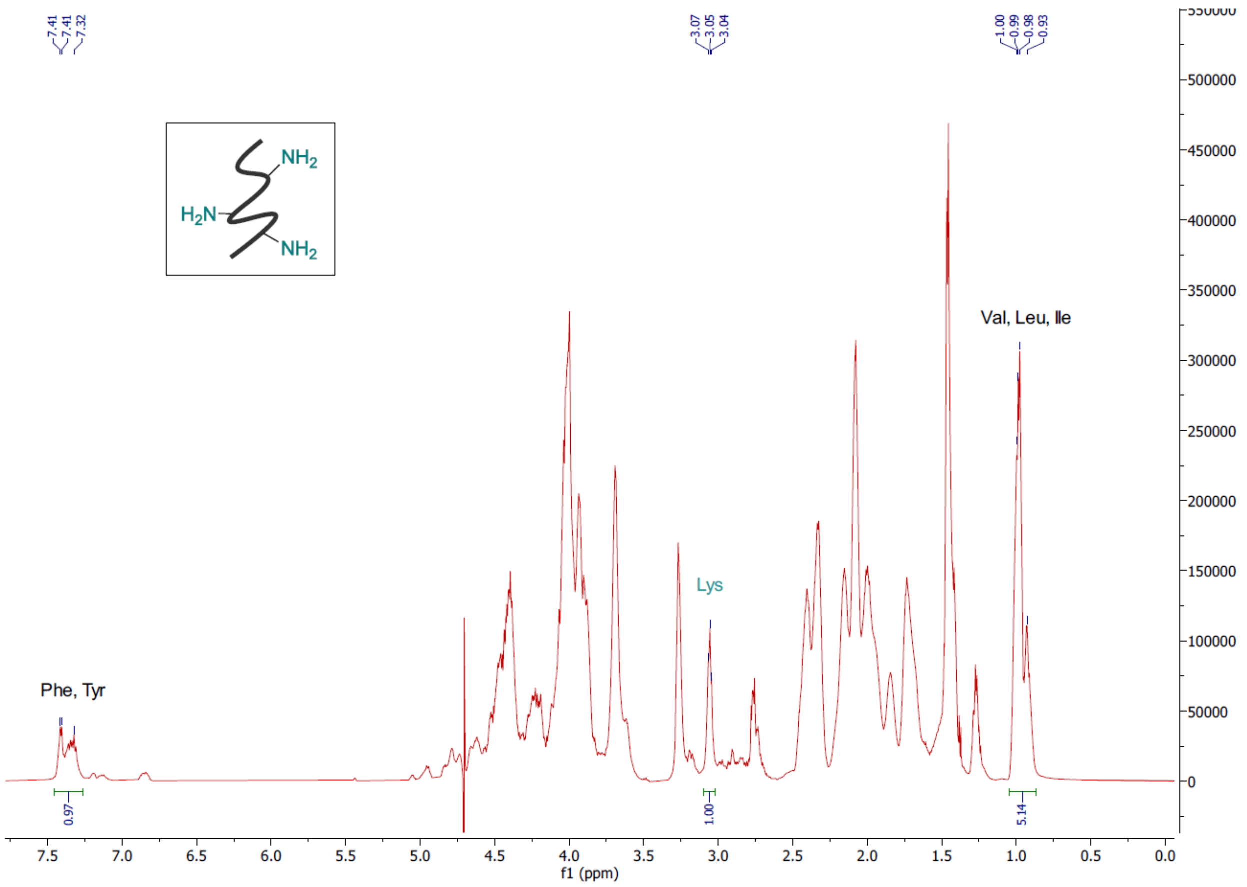

Figure S2: ^{1}H-NMR spectrum of the unfunctionalized Gelatin Medella Pro. (600 MHz, D_2O) δ: 7.41 – 7.32 (m, Phe, Thr), 6.30 (m, -CHa=CHb-), 6.24 (m, -CHa'=CHb'-), 5.97 (m, -CHa=CHb-), 3.07 – 3.04 (m, Lys), 1.00 – 0.93 (m, Val, Leu, Ile).

Table S1: Design of the DoEs and the resulting storage moduli (responses) for three different crosslinkers: DTT crosslinker (n=11), DTT:IKVAV crosslinker (n=11) and DTT:YIGSR crosslinker (n=11).

			DTT	DTT:IKVAV	DTT:YIGSR
**Nr.**	**GelNB [v/w%]**	**Crosslinker [mM]**	**Storage modulus *G'* [Pa]**	**Storage modulus *G'* [Pa]**	**Storage modulus *G'* [Pa]**
1	4.50	4.14	758	960	672
2	5.75	3.33	1342	1302	851
3	7.00	8.04	3023	2804	1760
4	7.00	4.14	1650	2070	1255
5	5.75	6.09	1913	1536	1011
6	7.38	6.04	2820	2850	1871
7	3.98	6.09	482	643	696
8	5.75	6.09	1748	1571	1170
9	5.75	8.85	2900	2081	1286
10	5.75	6.09	1698	1728	1035
11	4.50	8.04	1016	704	729

Table S2: Model statistics for the storage modulus.

Crosslinker	R^2	R^2_{adj}	Q^2	*p*-value	Reproducibility
DTT	0.94	0.91	0.79	1.35×10^{-4}	0.98
DTT:IKVAV	0.98	0.97	0.94	3.10×10^{-6}	0.98
DTT:YIGSR	0.98	0.96	0.90	5.57×10^{-5}	0.96

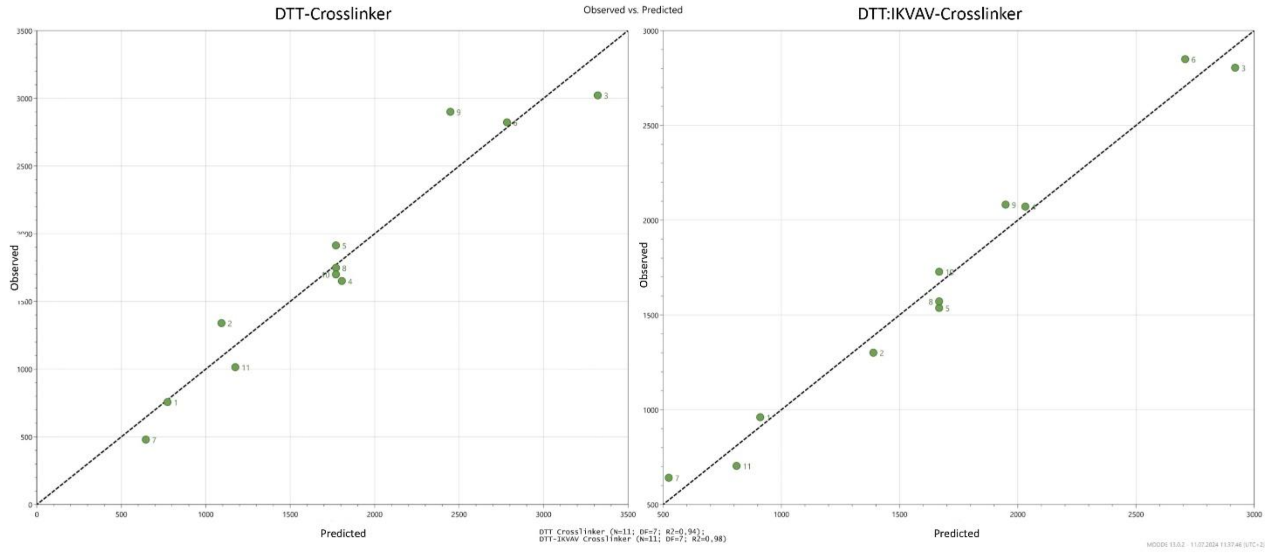

Figure S3: Observed vs. Predicted plots of both DoEs for the hydrogel stiffness response. DTT-Crosslinker: N=11; DF=7; R^2=0.94, *p*-value 1.35 x 10^{-4}, reproducibility 0.98. DTT:IKVAV-Crosslinker: N=11, DF=7; R^2=0.98, *p*-value 3.10 x 10^{-6}, reproducibility 0.98.

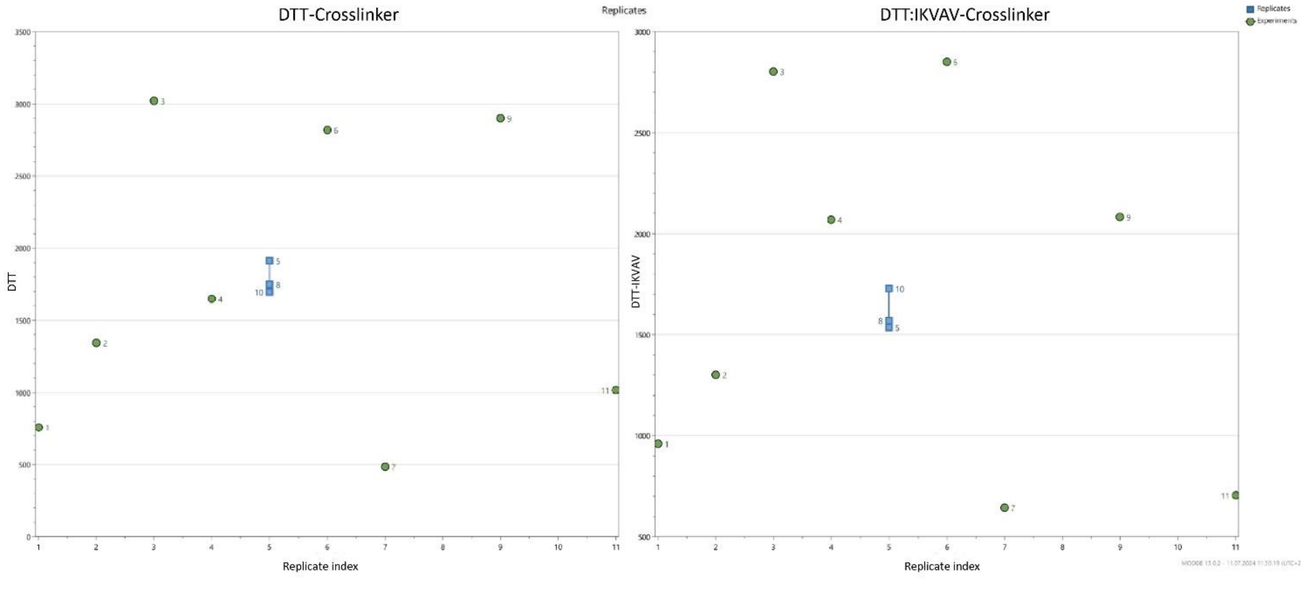

Figure S4: Reproducibility plots of both DoEs.

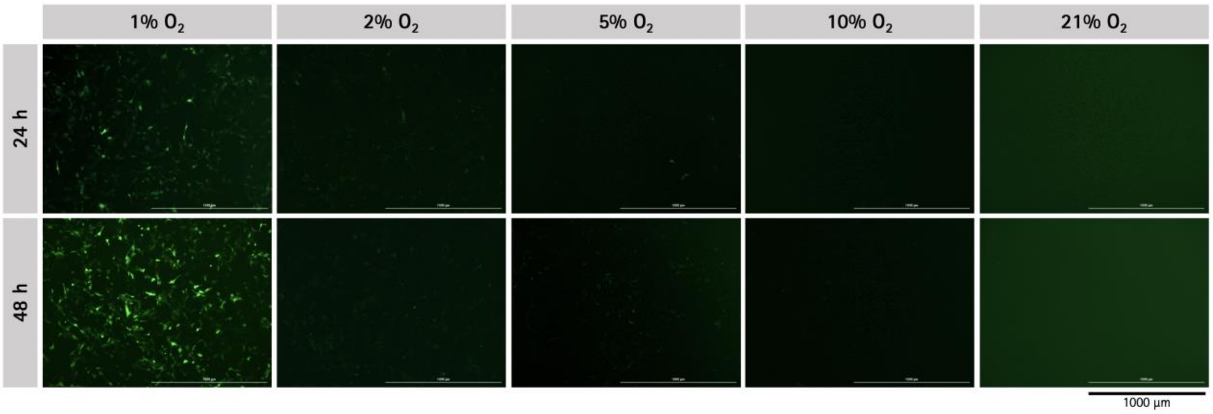

Figure S5: Comparison of ReNcell®CX-HRE3 fluorescence signal in 2D cell culture after 24 h and 48% cultivation in different oxygen concentrations (1%, 2%, 5%, 10%, 21%). Fluorescence microscopic analysis. Seeding density 2.1 x 10^4 cells/cm^2.

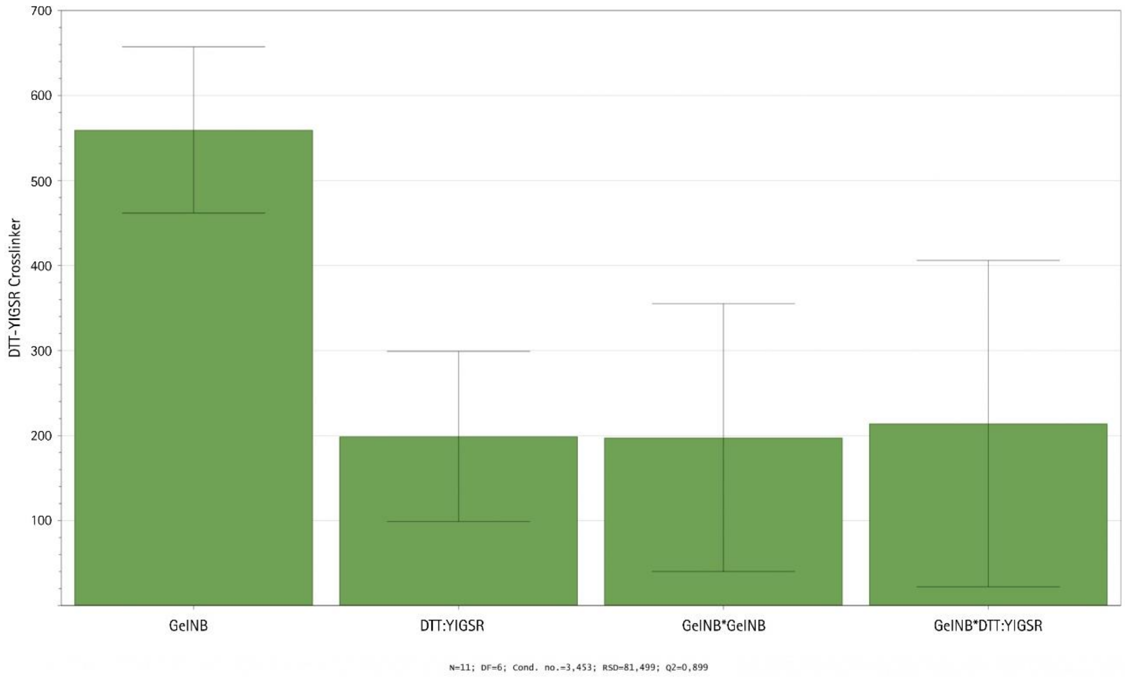

Figure S6: Coefficient plots for DoE with DTT:YIGSR as crosslinker molecule and its effect on the hydrogel stiffness response. Parameters not shown are those that included zero and therefore had no significant effect on the stiffness. GelNB [% (w/v)], DTT:YIGSR 1:2 [mM]. n=11, DF=6, $p < 0.05$.

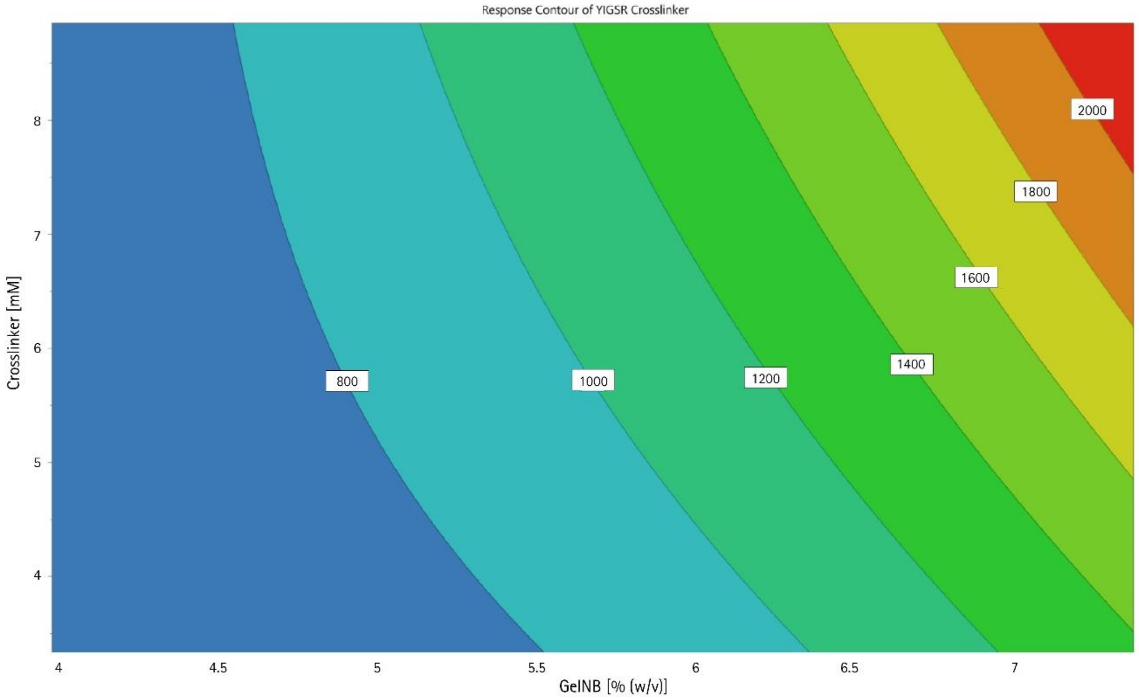

Figure S7: Response contour plot for the storage modulus response as a result of GelNB concentration and crosslinker DTT:YIGSR concentration.

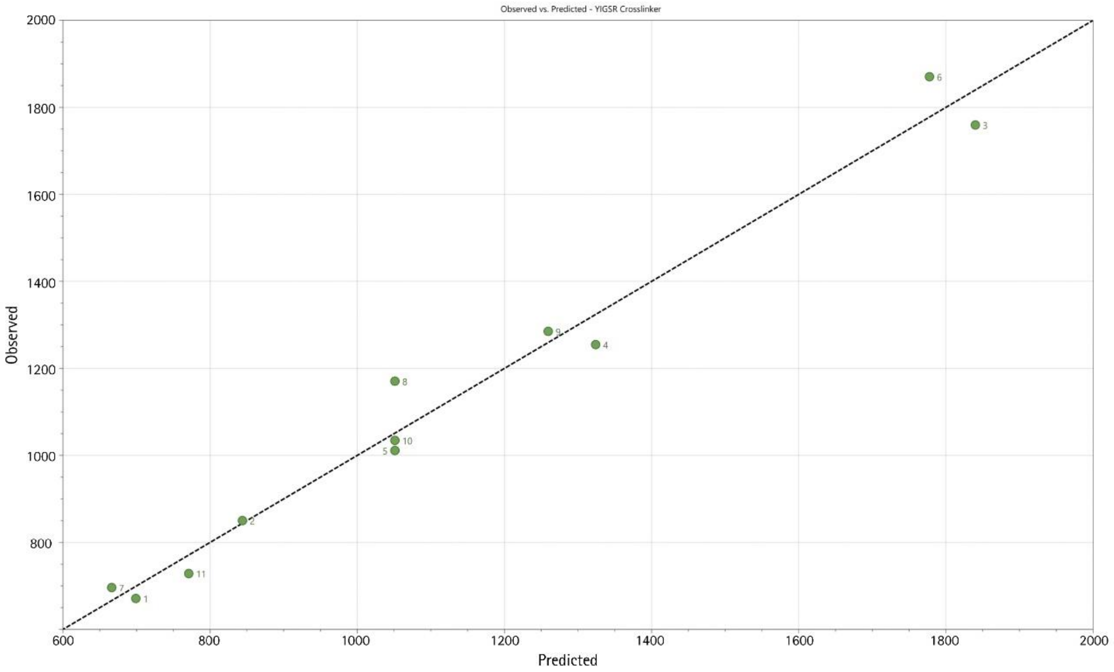

Figure S8: Observed vs. Predicted plots of DTT:YIGSR DoE for the hydrogel stiffness response. DTT:YIGSR-Crosslinker: N=11; DF=6, R^2=0.98, *p*-value 5.57 x 10^{-5}, reproducibility 0.96.

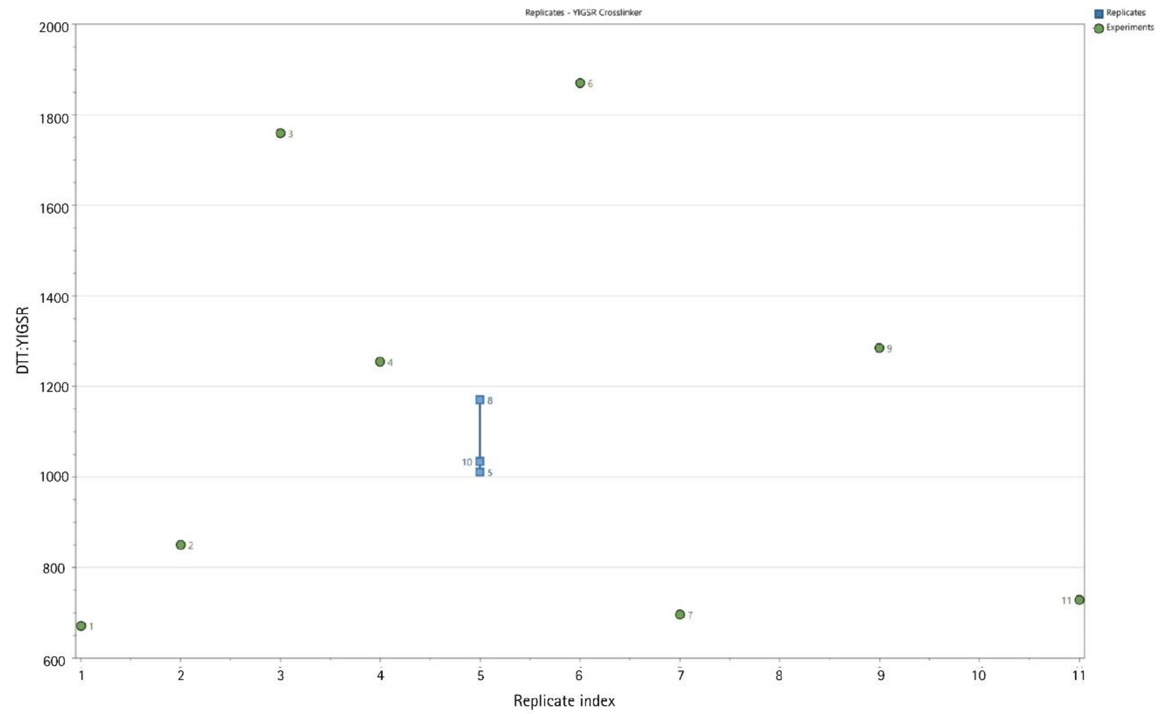

Figure S9: Reproducibility plot of DTT:YIGSR DoE.

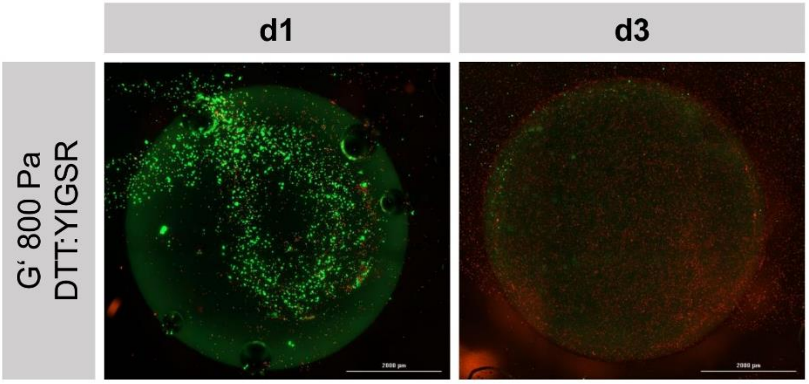

Figure S10: Fluorescence microscopic images ReNcell®CX cell viability after one and three days of cultivation (37 °C, 5% CO_2) in one GelNB hydrogel composition with DTT:YIGSR crosslinker. GelNB 4.53% (w/v), 2.196 mM DTT, 4.391 mM YIGSR. Encapsulated cell concentration 1 x 10^6 cells/mL. Hydrogel was dissolved on day 3. Green: Calcein-AM staining (viable cells), Red: Propidium iodide staining (dead cells).
